# Comparative Analysis of GnRH Pulse Generator Activity in Intact and Gonadectomized Male and Female Mice

**DOI:** 10.1210/endocr/bqaf099

**Published:** 2025-06-20

**Authors:** Bryan Chang, Ellen Gabrielle Wall, Allan Edward Herbison, Su Young Han

**Affiliations:** Department of Physiology, Development and Neuroscience, University of Cambridge, Cambridge BCB2 3EG, UK; Department of Physiology, Development and Neuroscience, University of Cambridge, Cambridge BCB2 3EG, UK; Department of Medicine, Division of Endocrinology, Diabetes and Hypertension, Brigham and Women’s Hospital and Harvard Medical School, Boston, MA 02115, USA; Department of Physiology, Development and Neuroscience, University of Cambridge, Cambridge BCB2 3EG, UK; Department of Physiology, Development and Neuroscience, University of Cambridge, Cambridge BCB2 3EG, UK; School of Psychology and Neuroscience, University of St. Andrews, St. Andrews KY16 9JP, UK

**Keywords:** GnRH, pulse generator, arcuate nucleus kisspeptin neurons, sex difference, gonadal steroid hormone

## Abstract

A subpopulation of kisspeptin neurons in the arcuate nucleus (ARN) of the hypothalamus functions as the GnRH pulse generator, driving the pulsatile secretion of LH from the anterior pituitary. Recent advances in in vivo GCaMP fiber photometry have allowed the direct measurement of ARN kisspeptin (ARN^KISS^) neuronal population activity in mice. In both sexes, ARN^KISS^ neurons display large, brief calcium activity episodes, termed synchronization episodes, each corresponding to a correlated LH pulse. Here we present quantitative and comparative analyses of calcium activity in these neurons and LH profiles in male and female mice, based on a combination of previously published and unpublished data.

Our findings reveal a significant sex difference in pulse generator frequency in intact mice, with males exhibiting slower and more stochastic synchronization episodes compared to females. Additional sex differences were noted in the profile of synchronization episodes. In gonadectomized mice, the synchronization frequency and the episode profiles became similar across sexes, indicating that gonadal steroids largely drive sex differences in the intact state. However, sex-specific differences in pulse frequency distributions persisted after gonadectomy, suggesting possible steroid-independent differences in the GnRH pulse generator. Sex differences in the LH pulse frequency and amplitude were observed in intact mice and were abolished following gonadectomy, highlighting the correlation between synchronization episodes and downstream hormonal signaling.

The GnRH neuronal network is fundamental in the control of the reproductive axis and fertility. Episodic GnRH secretion is required for functional gonadotropin secretion (1), and abnormalities in pulse frequency are associated with disorders of ovulation and infertility in women such as polycystic ovarian syndrome and hypothalamic amenorrhea (2). The arcuate nucleus (ARN) kisspeptin (ARN^KISS^) neurons are now recognized as the GnRH pulse generator (2). While early multiunit recordings demonstrated a correlation of ARN neuronal activity with LH pulses (3-6), the identity of neurons within the ARN had not been known. However, the crucial role of kisspeptin was discovered through the discovery of humans with *Kiss1r* mutations exhibiting abnormal LH pulses and hypogonadism (7), and *Kiss1r* KO mice having an absence of LH secretion, which could be rescued by returning *Kiss1r* expression in GnRH neurons (8). Over the last few years, it has become clear that ARN^KISS^ neurons are both sufficient and necessary for pulsatile secretion of LH in male and female mice (9) and that this population of neurons indeed exhibits synchronized episodes (SEs) of activity correlated with LH pulses (9-14). Although the role of ARN^KISS^ neuron synchronization in LH pulse generation has been demonstrated in many species (11, 15, 16), a direct comparison of their characteristics between males and females has not been provided to date. Taking advantage of the in vivo GCaMP fiber photometry in freely moving mice that enabled chronic measurements of synchronized activity of ARN^KISS^ neurons in both males (11) and females (13, 17), we provide quantitative and comparative analyses of the activity patterns of ARN^KISS^ neurons and the role of gonadal steroid hormones in male and female mice.

## Methods and Materials

### Animals

Adult male and female heterozygous KISS1-Cre^+/−^ mice (mixed 129S6Sv/EvC57BL6 genetic background) (11, 13, 17) were housed under 12:12 hours light/dark cycle (lights on: 6:00 Am in Otago, 7:00 Am in Cambridge) with ad libitum access to food and water. All experiments were approved by either the University of Otago Animal Welfare and Ethics Committee or the University of Cambridge Animal Welfare and Ethics Review Body under UK Home Office license P174441DE.

### Stereotaxic Injections of Adeno-associated Virus

As described previously (11, 13, 17), mice were anaesthetized with 2% isoflurane and placed in a stereotaxic frame with analgesia [either carprofen (5 mg/kg, subcutaneous [s.c.]) or meloxicam (5 mg/kg, s.c.) and buprenorphine (0.05 mg/kg, s.c.)] Cre-dependent adeno-associated virus (AAV) encoding GCaMP6s (AAV9-CAG-FLEX-GCaMP6s-WPRE-SV40, 1.3 × 10^13^ mol/mL; University of Pennsylvania Vector core, Philadelphia, PA, now Addgene, cat. no. 100842; 1 μL) was bilaterally injected into the ARN, and a 400-μm diameter optical fiber was then implanted directly above the ARN. Following surgery, mice were handled daily and habituated to the fiber photometry setup for at least 3 weeks.

### Fiber Photometry, Blood Sampling, and Pulsatile LH Assay

Fiber photometry recordings were undertaken to record GCaMP fluorescent signals from undisrupted, freely behaving mice in their home cage between 4 to 12 weeks after surgery. The recording conditions were consistent among all datasets. Recordings were made using a scheduled 5 seconds/20 seconds on/off mode for 4 hours in males and in females on each of the 4 stages of the estrous cycles. Additional recordings were performed in 10 Hz continuous mode, for 2 to 6 hours and 1 hour, in intact and gonadectomized (GDX) mice, respectively, to capture 1 or more synchronization episodes (SEs) in a high temporal resolution. These recordings in intact females were taken during all estrous cycle stages except for estrus, as it was previously shown that SE profiles do not vary between different cycle stages (13).

To examine the relationship between SEs and pulsatile LH secretion, separate photometry recordings sessions were conducted, concurrently with serial tail-tip blood sampling (18). In intact males, 3 μL samples were taken every 3 to 6 minutes for a 120- to 240-minute duration (11), and in intact females, 4 μL samples were taken every 5 to 10 minutes for a 100- to 150-minute duration in diestrus (13). Under GDX conditions, 3 μL samples were collected every 3 minutes in males and every 5 minutes in females during the 60- to 90-minute recording period (11, 17). The LH level was assessed using the ultrasensitive LH ELISA of Steyn and colleagues (19). The reagents used for the LH ELISA were as follows: a capture antibody (monoclonal antibody, antibovine LH β subunit, 518B7, University of California; RRID: AB_2665514), a standard (mouse LH reference preparation, AFP-5306A, National Institute of Diabetes and Digestive and Kidney Diseases, National Hormone and Pituitary Program), and a detection antibody (polyclonal antibody, rabbit LH antiserum, AFP240580Rb, National Institute of Diabetes and Digestive and Kidney Diseases, National Hormone and Pituitary Program, RRID: AB_2665533).

### Analysis of the Pulse Generator Activity in Mice

**Figure 1.**
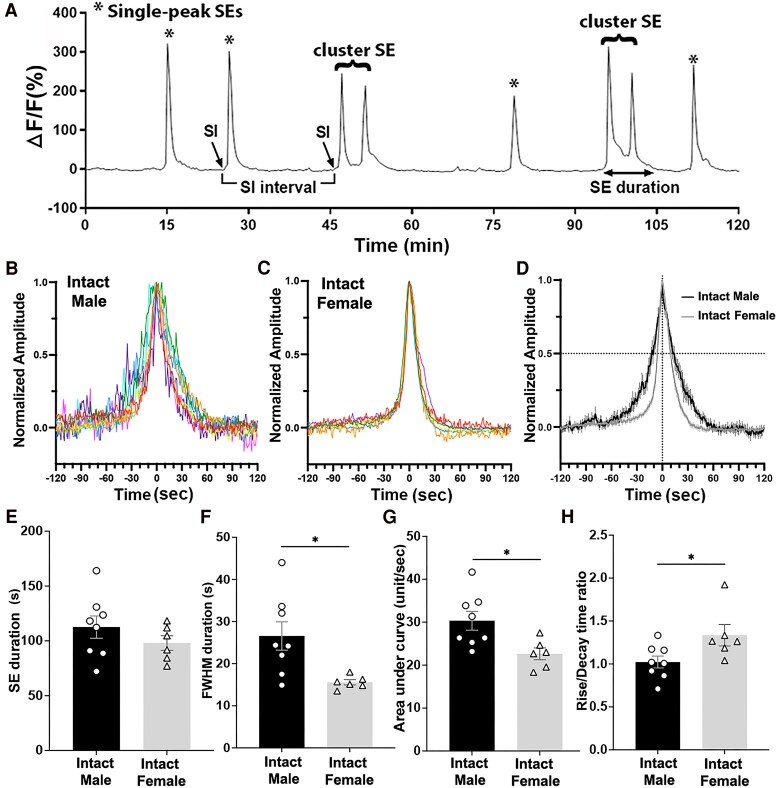
(A) An example trace of 120-minute fiber photometry recording from arcuate kisspeptin neurons in a gonadectomized male mouse, indicating the parameters used in the study. Single-peak SEs (*), cluster SEs comprised of multiple peaks, SI points, and SE duration are indicated. (B, C) GCaMP6 photometry recordings of individual SEs in intact mice captured in a 10-Hz continuous acquisition mode. Normalized traces from individual male (B, n = 8) and female (C, n = 6) mice, each with a peak amplitude set to 1.0, are displayed in different colors. Note in females SEs from all stages of the estrus cycle were used since it was previously shown not to vary in different stages (13). (D) Mean ± SEM profiles of male (black) and female (grey) mice are overlayed. The vertical dashed line at time 0 indicates the time of the peak. The horizontal dashed line indicates the normalized amplitude of 0.5, where the FWHM values are taken. (E) Mean ± SEM of SE durations in intact males and females are not different (*P* = .35, Mann–Whitney). (F) FWHM duration is significantly higher in intact males compared to females (*P = .013, Mann–Whitney). (G) Area under the curve within an SE (±120 seconds around the peak) is significantly higher in males compared to females (*P = .013, Mann–Whitney). (H) The ratio of rise time (baseline to peak) to decay time (peak to baseline after peak) is significantly higher in females (*P = .021, Mann–Whitney). Abbreviations: FWHM, full-width half maximum; SE, synchronization episode; SI, synchronization initiation.

Data were collected from adult male and female mice sourced from published (11, 13, 17) and unpublished studies. The whole dataset of recorded ARN^KISS^ neuron activity was reanalyzed by 1 person not involved in either of the experiments to avoid interexperimenter subjectivity in determining the parameters. A robust linear regression was applied to correct for baseline photobleaching during long-term imaging. Next, the raw fluorescence data was converted to ΔF/F (%) calculated using ΔF/F (%) = (F−F^basal^)/F × 100. A large, abrupt increase in calcium signal with a duration of ∼80 to 100 seconds (Fig. 1A), defined as an SE, is a typical activity pattern of ARN^KISS^ neuron populations. The peak amplitude of each SE varied considerably between animals with a range of 8% to 900% ΔF/F, likely due to variability in the placement of the fiber optic. Therefore, the SE amplitude during the GDX and intact states was expressed as a ratio within the same animal. The frequency of the pulse generator was measured by calculating intervals between each synchronization initiation (SI) point (Fig. 1A). The SI point was defined as the point at which ΔF/F begins to exhibit a sustained increase in signal over at least 2 consecutive data points, each representing the average of a 5-second recording period, leading to a SE. This point was manually identified by a researcher. In GDX mice, some SEs exhibited multiple peaks occurring in succession before returning to baseline as previously reported (11, 17); hence, SEs were classified as either single-peak SEs or cluster SEs (Fig. 1A). The first SI points (Fig. 1A) of cluster SEs were used to analyze SI intervals in GDX mice. The number of peaks and the total duration of both single-peak and clustered SEs were measured over the 4-hour recording period and presented as the mean for each animal. To account for possible sex differences in the “free-running” patterns of the pulse generator activity, the SE dynamics were compared between long-term (3 weeks or longer) GDX males and females.

### Statistical Analyses

All statistical analyses were performed in Prism 10 (GraphPad Software Inc.). All values are expressed as mean ± SEM unless otherwise stated, and the significance is defined as *P < .05, **P < .01, or ***P < .001. The Mann–Whitney test was used for male and female comparisons, and the Kruskal–Wallis test was used for comparing SI intervals between intact females throughout estrous cycles and males. The Kolmogorov frequency test was employed to assess the distribution of synchronization episode frequencies.

## Results

### Pulse Generation in Intact Male and Female Mice

#### Detailed profiles of individual synchronization episodes

Recordings using a continuous 10-Hz sampling rate capturing 1 to 4 SEs in each mouse were made to assess detailed profiles of individual SEs in male (n = 11 SEs in 8 mice) and female (n = 20 SEs in 6 mice). The SEs in males and females both showed a near symmetrical shape (Fig. 1B–1D). The total duration of an SE was not significantly different between males and females (113 ± 10 seconds and 98 ± 7 seconds, respectively; *P* = .35, Mann–Whitney; Fig. 1E). However, both the incline and decline of the SEs were sharper in females (Fig. 1B–1D), demonstrated by a significantly larger full width at half maximum (FWHM) in males (27 ± 3 seconds and 16 ± 1 seconds in males and females, respectively, *P* = .013, Mann–Whitney; Fig. 1F). In addition, the area under the curve value for the duration of an SE was 30 ± 2 unit/sec in males, which was 23% larger than the 23 ± 1 unit/sec in females (*P* = .013, Mann–Whitney; Fig. 1G). A difference in the dynamics of synchronization was observed, with the rise time to decay time ratio around the peak being significantly higher in females (1.3 ± 0.1) compared to males (1.0 ± 0.1; *P* = .021, Mann–Whitney test; Fig. 1H). The ratio of 1.0 in males reflects a symmetrical timescale for the rise to and fall from the peak, whereas the ratio of 1.3 in females indicates a longer rise time compared to the decay time duration.

#### Frequency and distribution patterns of pulse generation in intact male and female mice

**Figure 2.**
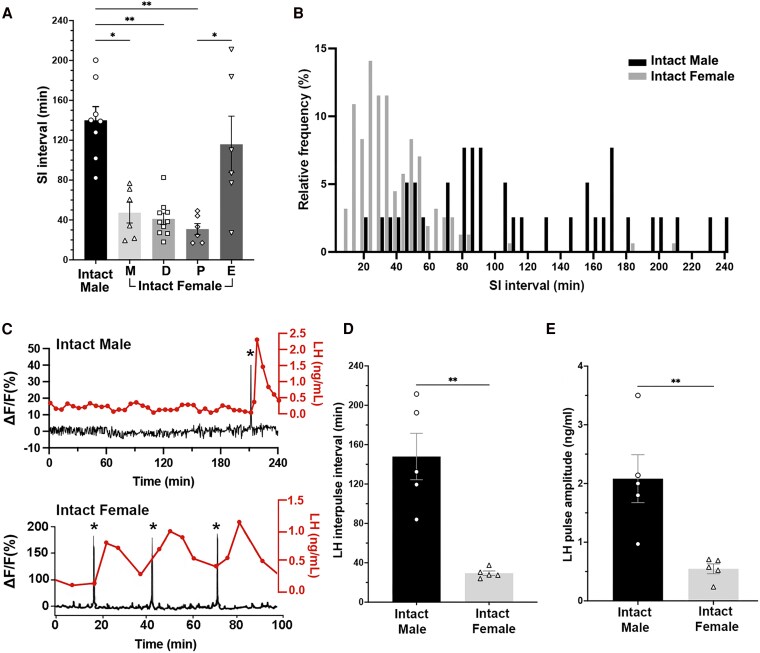
(A) Mean ± SEM SI intervals in intact males (n = 8) and females across the estrous cycle (M: n = 6; D: n = 10; P: n = 6; E: n = 6). There is a significant increase in the SI interval in estrus compared to other phases of the cycle (Kruskal–Wallis, *P* < .001). Except for estrus, the female SI interval is significantly lower than in males. The SI interval is significantly prolonged in estrus compared to proestrus, with a trend toward further increases in metestrus and diestrus. Significant differences are indicated with asterisks. *P* = .046, male vs M; *P* = .0082, male vs D; *P* = .0016, male vs P; *P* = .048, P vs E, Dunn's multiple comparisons test. (B) SI interval distribution histogram in intact males (black) and females (grey) overlayed, data presented in 5-minute bins. Numbers on the x-axis indicate bin centers. The SI interval distribution in male and female gonadectomized mice is significantly different (*P* < .001, Kolmogorov–Smirnov D = 0.73). (C) Representative examples from a male (upper panel) and a female (lower panel) mouse, showing perfect correlation between SEs (*) and LH pulses (aligned immediately above the photometry trace). Adapted from Han et al., Endocrinology, 2019 (male), and McQuillan et al., Endocrinology, 2019, by permission of Oxford University Press. (D) LH interpulse interval is significantly higher in intact males than in females (n = 5 each group, **P < .01, Mann–Whitney). (E) LH pulse amplitude is significantly higher in intact males than in females (n = 5 each group, **P < .01, Mann–Whitney). Abbreviations: D, diestrus; E, estrus; M, metestrus; P, proestrus; SE, synchronization episode; SI, synchronization initiation.

The interval between each SI was measured to determine the pulse generator frequency during the light-on phase (4-hour recording starting 2-3 hours after light-on) in intact males and females in 4 stages of the estrous cycle. In males, the pulse generator frequency was 140.1 ± 15.8 minutes (n = 8). This was significantly slower than that of females during metestrus (47.4 ± 10.4 minutes, *P* = .046; n = 6), diestrus (41.1 ± 5.7 minutes, *P* = .0082; n = 10), and proestrus (30.9 ± 5.6 minutes; *P* = .0016; n = 6) (Fig. 2A; *P* = .003, Kruskal–Wallis followed by Dunn's post hoc test). However, the male pulse generator frequency was similar to that of estrous females (*P* > .9999; Fig. 2A), as the frequency in estrous females greatly slowed to 115.5 ± 28.5 minutes (*P* = .51 vs metestrus, *P* = .22 vs diestrus, *P* = .048 vs proestrus; n = 6, Dunn's post hoc test; Fig. 2A).

The distribution pattern of male and female SI intervals were determined by plotting each individual SI interval measured. The SI interval distribution was significantly different between males and females (Kolmogorov–Smirnov test, *P* < .001, D = 0.73): males showed a very wide range of SI intervals between 20 and 238 minutes (n = 47 SI intervals in 8 mice; Fig. 2B), with no obvious modal distribution pattern as reported previously (11). In contrast, female SI intervals across all estrous cycles have a more clustered distribution with a slight rightwards skew, ranging between 9 and 211 minutes with 37% of SEs occurring with intervals between 22.5 and 37.5 minutes (n = 164 SI intervals in 10 mice; Fig. 2B). Thus, in an intact state, the pulse generator operates at a more consistent and faster pace in females compared to males.

#### Male and female pulse generator activity shows tight correlation to LH pulses

We have previously shown that SEs have a near-perfect correlation to a pulse of LH (11, 13, 14, 17). The frequency and amplitude of the LH pulses correlated to photometry recordings were compared between intact males (n = 5) and diestrous females (n = 5) (Fig. 2C). The average interpulse interval in females was 29 ± 3 minutes which was significantly shorter than that in intact males at 148 ± 24 minutes (*P* = .008, Mann–Whitney; Fig. 2D), indicating faster pulse generation in females. Additionally, the amplitude of LH pulses was significantly lower in females (0.6 ± 0.1 ng/mL; *P* = .008, Mann–Whitney) than in males (2.0 ± 0.5 ng/mL) (Fig. 2E).

### SEs in Long-term GDX Male and Female Mice

Gonadal steroid hormones provide negative feedback to LH pulsatility. Pulse generator activity following the removal of gonads has been reported in male (11) and female mice (17). Gradual changes in SE parameters occur with time following gonadectomy, as previously described (11, 17). To account for possible sex differences in the “free-running” patterns of the pulse generator activity, the SE dynamics were compared between long-term (3 weeks or longer) GDX males and females.

#### SE amplitude

**Figure 3.**
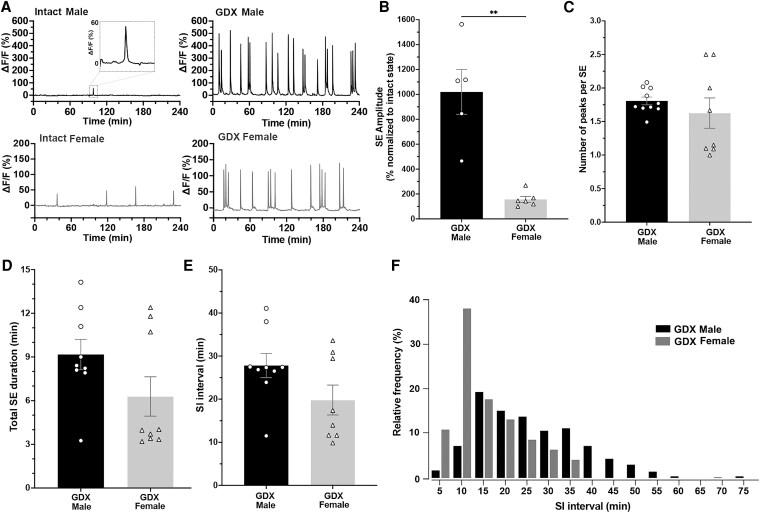
(A) Representative 4-hour fiber photometry recordings of arcuate kisspeptin neurons in intact (left) and long-term GDX (right) male (upper) and female (lower) mice. A notable increase in SE amplitude is observed, accompanied by the emergence of cluster SEs in both sexes. (B) Mean ± SEM amplitudes in SE GDX male (n = 5) and female mice (n = 6). The amplitudes of SE in GDX mice were normalized to their respective intact state value and displayed as a percentage. For example, a value of 100% would indicate no change after GDX, whereas values above or below 100% represent increases or decreases in SE amplitude, respectively. The GDX amplitude in males is significantly higher than that in females (**P = .004, Mann–Whitney). (C) Number of peaks per SE is not different between GDX male (n = 8) and female (n = 8) mice (*P* = .33, Mann–Whitney). (D) Total SE duration is not different between GDX male (n = 8) and female (n = 8) mice (*P* = .22, Mann–Whitney). (E) Mean SI interval is not different between GDX male (n = 8) and female (n = 8) mice (*P* = .32, Mann–Whitney). (F) A distribution histogram of the SI interval in GDX males (black) and females (grey) overlayed; data presented in 5-minute bins. Numbers on the x-axis indicate bin centers. The SI interval distribution in male and female GDX mice is significantly different (*P* < .001, Kolmogorov–Smirnov D = 0.447). Abbreviations: GDX, gonadectomized; SE, synchronization episode; SI, synchronization initiation.

The amplitude of SEs was increased by gonadectomy in both sexes (Fig. 3A). To compare the relative increase between males and females, the SE amplitudes in GDX males and females were normalized, intact values in the same male (n = 5) and female (n = 6) mice. This ensures direct comparison between the intact and GDX states is possible despite the individual variability in the absolute ΔF/F due to differences in probe placement. Surprisingly, the amplitude of SEs in males increased by 850% after gonadectomy compared with only 160% in females (*P* = .004, Mann–Whitney; Fig. 3B).

#### Cluster SEs and total SE duration

One of the prominent changes in the pulse generator activity following gonadectomy is the emergence of a cluster of SEs, whereby multiple peaks occur within an episode (Figs. 1A, 3A). The emergence of clusters of SEs has been observed within a few days post-gonadectomy and persists in the long term for both males and females (11, 17). To examine sex differences in synchronization stability in a steroid-free milieu, the mean number of peaks per SE cluster and the SE cluster duration were compared between the long-term GDX males and females. Interestingly, the mean number of peaks within an SE cluster was similar in males and females (1.76 ± 0.06, n = 9 and 1.59 ± 0.20, n = 8 respectively, *P* = .25, Mann–Whitney; Fig. 3C). Therefore, the total SE duration for each animal was calculated by combining both single and cluster SEs, which were characterized by prolonged duration over several minutes, in both males (9.1 ± 2.1 minutes) and females (6.2 ± 1.6 minutes). The total SE duration in GDX mice was not different between sexes (*P* = .22, Mann–Whitney; Fig. 3D).

#### SI interval

The absence of gonadal hormones leads to faster pulse generator activity, as indicated by the increased frequency of LH pulses in GDX male and female mice (18). The direct photometry measurements of the ARN^KISS^ neurons confirm that the frequency of pulse generation speeds up within a few days of gonadectomy and then persists over the long term (11, 13, 17). To assess sex differences in the pulse generator frequency in a steroid-free milieu, the mean SI intervals of the long-term GDX males and females were compared. Unlike in the intact state, the mean SI interval in GDX males was not different from that in GDX females (28.3 ± 2.8 and 20.0 ± 3.5 minutes, respectively, *P* = .32, Mann–Whitney; Fig. 3E).

In addition to a faster frequency of operation, the more stochastic pattern of SEs observed in intact male mice disappeared in the GDX state, with mice exhibiting more regular SE patterns (Fig. 3F). Nearly 20% of SI intervals occurred between 12.5 and 17.5 minutes (Fig. 3F). In females, the right-skewed distribution remained as in intact state; however, the largest proportion of SI intervals was between 8.5 and 12.5 minutes, indicating more frequently occurring synchronizations. The distribution range was 5 to 36.9 minutes in females, whereas males showed a slightly larger range of synchronization intervals between 3.6 and 76.5 minutes. To compare SI interval distributions, we employed Kolmogorov–Smirnov testing, which showed that the SI interval distributions in male and female GDX mice remain significantly different (*P* < .001, Kolmogorov–Smirnov D = 0.447).

#### Profiles of individual single-peak SEs

**Figure 4.**
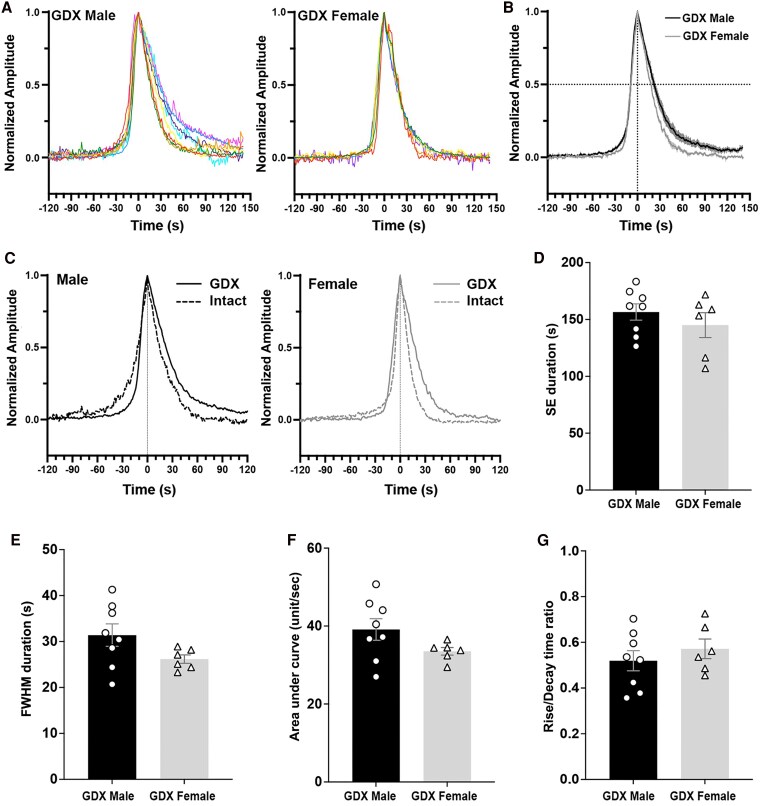
Profiles of single-peak SEs in GDX mice captured by GCaMP photometry recordings in 10-Hz continuous mode. (A, B) Normalized traces from individual male (left) and female (right) mice, each with a peak amplitude set to 1.0, are displayed in different colors. (B) Mean ± SEM profiles of male (black, n = 8) and female (grey, n = 6) mice are overlayed. Vertical dashed line at time 0 indicates the time of the peak. The horizontal dashed line indicates the normalized amplitude of 0.5, where the FWHM values are taken. (C) Mean of SE profiles stratified by their respective gonadal status (GDX in solid lines and intact in dashed lines) overlayed, for both males (left, black) and females (right, grey). (D) Mean ± SEM of SE durations in GDX males and females (*P* = .41, Mann–Whitney). (E) FWHM duration is not different between GDX males and females (*P* = .12, Mann–Whitney). (F) Area under the curve within an SE is not different between GDX males and females (*P* = .11, Mann–Whitney). (G) The ratio of rise time (baseline to peak) to decay time (peak to baseline) is not different between GDX males and females (*P* = .41, Mann–Whitney). Abbreviations: FWHM, full-width half maximum; GDX, gonadectomized; SE, synchronization episode; SI, synchronization initiation.

Gonadectomy changes the dynamics of the single-peak SE profile in male mice (11), resulting in a faster incline. Although the profiles of SEs in intact females have been reported (13), gonadectomy-induced changes in the single-peak SE profile in female mice had not been analyzed. To investigate and compare the roles of gonadal steroid hormones in determining SE dynamics in male and female mice, the individual single-peak SE profiles of long-term GDX male and female mice were evaluated. Recordings of up to 3 hours using 10-Hz continuous mode were made to assess the detailed profiles of individual single-peak SEs in GDX males (n = 28 SEs in 8 mice) and GDX females (n = 20 SEs in 6 mice). In both male and female mice, gonadectomy resulted in a common change in the SE profile to a more right-skewed shape, unlike the near-symmetrical shape observed in the intact state (Fig. 1B–1D, Fig. 4A–4C). The full duration of a single-peak SE in GDX mice was not significantly different in males (157 ± 7 seconds) and females (145 ± 11 seconds) (*P* = .41, Mann–Whitney; Fig. 4D). Similarly, no sex differences were observed in FWHM values (31 ± 2.4 seconds in males vs 26 ± 0.9 seconds in females, *P* = .115, Mann–Whitney; Fig. 4E) or in the area under the curve (39 ± 2.8 unit/sec in males vs 34 ± 1.0 unit/sec in females, *P* = .11, Mann–Whitney; Fig. 4F). The rise/decay time ratio was calculated as a measure of SE profile skewness, where the ratio of 1.0 would indicate a symmetrical shape. In both males and females, the ratio was shorter than 1.0 (0.52 ± 0.04 in males and 0.57 ± 0.04 in females), indicating faster incline. The rise/decay time ratio was not different between males and females (*P* = .41, Mann–Whitney; Fig. 4G).

#### Profiles of LH pulses in GDX mice

**Figure 5.**
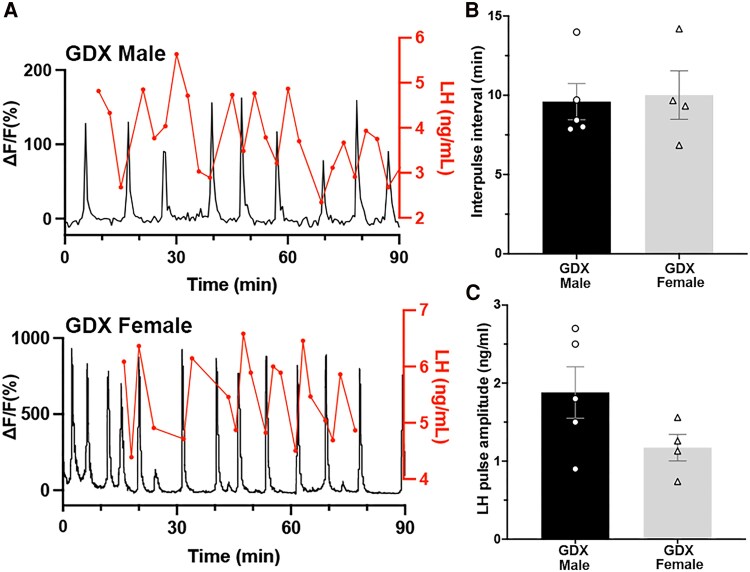
Pulsatile secretion of LH in GDX males and female mice. (A) Representative examples from a male (upper panel) and a female (lower panel) mouse, showing near-perfect correlation between SEs and LH pulses (aligned immediately above the photometry trace). (B) LH interpulse interval is not different between males (n = 5) and females (n = 4) (*P* = .5, Mann–Whitney). (C) LH pulse amplitude is not different between males (n = 5) and females (n = 4) (*P* = .76, Mann–Whitney). Abbreviations: GDX, gonadectomized; SE, synchronization episode.

LH pulses in GDX mice occur more frequently than in the intact state, often exhibiting a tooth saw shape with smaller amplitude (18). This is due to the faster operation of the pulse generator activity after gonadectomy (11, 17) (Fig. 5A). A direct comparison of LH pulsatility between GDX males and females was made from the LH dataset that had been correlated with calcium activity measured by photometry. The interpeak interval of LH pulses in GDX males (n = 5) was 9.5 ± 1.2 minutes, which was similar to that in females (10.0 ± 1.5 minutes, *P* = .76, Mann–Whitney, n = 4; Fig. 5B). The amplitude of LH pulses, defined by the difference between peak and the previous nadir value, showed no difference between males (n = 5) and females (2.1 ± 0.5 ng/mL vs 1.2 ± 0.2 ng/mL, respectively, *P* = .5, Mann–Whitney, n = 4; Fig. 5C).

## Discussion

We report here a detailed, quantitative analysis of the GnRH pulse generator synchronization patterns in adult male and female mice measured by GCaMP fiber photometry. Both males and females exhibit large, abrupt population increases in calcium activity, termed SEs, that generate a pulse of LH. In comparing this population activity in males and females, we find a marked sex difference in the pulse generator frequency in intact mice, with a tighter SI interval distribution in females. Differences in the dynamics of individual SEs are also observed. In GDX mice, the sex difference in pulse generator frequency is largely removed, although differences in SI interval distribution remain. No sex differences in the dynamics of individual SEs are observed in GDX mice.

### The Use of In Vivo Fiber Photometry for Studying Pulse Generator Activity

Early multiunit recordings established a correlation of ARN neuronal activity with LH pulses (6); the identity of neurons within the ARN remained unknown. Advances in genetically encoded calcium indicators, such as GCaMP, combined with Cre-Lox-based transgenic mouse models, have positioned GCaMP fiber photometry as a powerful tool for detecting calcium activity in ARN^KISS^ neurons in vivo (11, 13). A key advantage of in vivo fiber photometry is its ability to capture the endogenous, synchronized activity that defines the GnRH pulse generator. Furthermore, because mice tolerate fiber photometry for extended periods, this technique enables within-subject comparisons, such as pre- and post-gonadectomy assessments, as demonstrated in this study. By utilizing Cre-dependent stereotaxic AAV injections into the arcuate nucleus of KISS1-Cre^+/−^ mice, GCaMP6 expression can be specifically localized to ARN^KISS^ neurons, ensuring targeted and reliable recordings. Notably, the number of ARN^KISS^ neurons is consistent between sexes (20, 21), and a comparable proportion of ARN^KISS^ neuron populations express GCaMP in both males and females (11, 13). Additionally, studies in acute brain slices have shown that GCaMP6 provides a highly reliable index of ARN^KISS^ neuronal firing (9). Individual ARN^KISS^ neurons also exhibit burst firing during population miniature SEs (12, 22), further validating GCaMP6 signals as an accurate reflection of synchronized neuronal activity. Hence, there is a high degree of confidence that GCaMP6 fiber photometry provides a robust and accurate assessment of ARN^KISS^ neuron population firing in vivo, making it an invaluable tool for studying neural dynamics across different physiological conditions and between sexes.

### Pulse Generator Activity in Intact Mice

Our primary finding is that the GnRH pulse generator activity patterns, measured by the frequency of SEs and its distribution of ARN^KISS^ neurons, is different in intact males and females. The female pulse generator activity, except in estrus, showed a more regular pattern of activity with a faster pace compared to males. This is also compatible with recent long-term recordings of female ARN^KISS^ neurons across the estrous cycle (23). The faster synchronization frequency in intact females is compatible with previous studies reporting shorter LH interpulse intervals of 38 to 60 minutes in diestrous female mice (18, 24) compared with ∼200 minutes in male mice (11, 25). The more frequent and regular LH pulsatility in female mice would be important for providing a dynamic range of signals required for ovary during a reproductive cycle. In contrast, in male mice, a slower minimum LH pulsatility every 2 to 3 hours may be sufficient for spermatogenesis, although the more infrequent LH pulses may be compensated by the higher amplitude in LH pulse achieved by a single synchronization, facilitated by a higher pituitary sensitivity to GnRH (26).

When examining the detailed dynamics of individual synchronization episodes, we observed that males exhibit a broader SE profile in the intact state, reflected by larger area under the curve and FWHM values. While the exact number of kisspeptin neurons recruited during an SE remains unknown, these findings suggest potential differences in recruitment dynamics that may underlie the observed sex-specific synchronization profiles. The broader synchronization window in males may result in a more prolonged and dispersed release of kisspeptin onto GnRH neuron dendrons, ultimately contributing to the higher LH pulse amplitudes observed in this study and elsewhere (11, 13, 26, 27). In contrast, faster recruitment of ARN^KISS^ neurons in females may drive sharper, more synchronized SEs, potentially optimizing the temporal precision of GnRH/LH release.

### The Pulse Generator Activity Is Similar in GDX Male and Female Mice

It appears that the fundamental operating mechanism of the pulse generator is similar in both male and female mice, and most of the observed sex differences in the intact state can be attributed to the action of gonadal steroids. This is supported by the removal of significant sex differences in SI frequency following gonadectomy, although subtle differences in the SI interval distribution persist, as will be discussed. Moreover, the emergence of clustered SEs in both males and females after gonadectomy suggests that circulating gonadal steroids play a key role in restraining both the initiation and the termination of synchronization episodes in both sexes. Given that gonadal steroids modulate the sensitivity of ARN^KISS^ neurons to coreleased neuropeptides like neurokinin B (NKB) and dynorphin, the enhanced synchronized activity in GDX mice could stem from increased NKB-mediated activation and reduced dynorphin-mediated suppression (28-32).

The dynamics of individual SEs in GDX males and females are also remarkably similar, with both sexes exhibiting a rapid increase followed by a slower decay of SEs compared to their intact state, as reflected in a similar rise time/decay time ratio. Interestingly, the onset of SEs was more rapid in GDX mice compared to intact animals, suggesting that circulating steroids normally delay ARN^KISS^ neuronal recruitment. Steroid feedback likely raises the activation threshold or enhances inhibitory tone, slowing synchronization onset; its removal thus leads to a more excitable ARN^KISS^ network and faster SE initiation. The frequent appearance of multiple peaks within single SEs in GDX mice, indicative of unstable initiation and termination and increased susceptibility to reactivation, further supports this view. Such dynamics may reflect heightened recruitment of ARN^KISS^ neurons, potentially mediated by altered glutamatergic, NKB, or dynorphin signaling (28-30); changes in gonadal hormone-sensitive gene expressions or afferent inputs (28, 33, 34); and/or modulation of ion channels (35). The functional significance of this remains unclear, though the synchronization profile may contribute to shaping the temporal dynamics of kisspeptin neuropeptide release. To determine how circulating steroids modulate the rapid sequential activation of individual ARN^KISS^, observed in GDX mice of both sexes (12, 14), single-cell resolution recordings in intact animals will be useful.

In GDX mice, we observed no significant sex differences in LH pulsatility rate or amplitude. Both males and females displayed an LH pulsatility rate of approximately 10 minutes, consistent with previous findings (9, 36, 37). However, this pulsatility rate contrasts with the higher mean SI intervals observed in this study, suggesting that the stress associated with LH sampling may interfere with the natural endogenous rate of pulse generator activity.

### Greater Repressive Effect of Gonadal Steroids on GnRH Pulse Generator in Males

One noteworthy observation is the significantly greater amplification of the SE height following gonadectomy in males compared to females. The minimum percentage increase in SE amplitude in GDX males was 450%, far exceeding the maximum observed in females (263%). Since relative changes in amplitude were measured, our ability to draw conclusions about absolute SE amplitude differences between sexes in the GDX state is limited. However, these findings strongly suggest that gonadal steroids exert a greater suppressive effect on SE amplitude and pulse frequency in males than in females, possibly through differences in gonadal hormone levels and their interaction with downstream signaling pathways.

The mechanisms underlying these sex differences in gonadal steroid regulation of pulse generator activity remain unclear. The interplay between gonadal steroids and their receptors is complex, and the extent to which steroids differentially modulate the pulse generator activity through different receptors in males and females remains to be fully understood. Estradiol levels are consistently lower in males than in females at any estrous stage (38), whereas testosterone levels are significantly higher in males (39). Kisspeptin neurons in both male and female rodents express estrogen receptor (ER)α and ERβ, with ERα expression being higher in females (39, 40), while ERβ is more abundant in males (41). Additionally, ARN^KISS^ neurons in both sexes express androgen receptors (42-44) and progesterone receptors (33, 45, 46). The ERα knockdown in female ARN^KISS^ neurons alters synchronization patterns to resemble those seen in the ovariectomized state (14), underscoring the critical role of ERα in steroid hormone modulation of pulse generator activity. However, whether the identical effect occurs in males remains unknown. Similarly, in females, the pulse generator activity is greatly suppressed during the estrus phase through the action of progesterone, and this suppression can be replicated by exogenous progesterone administration (13). While evidence suggests that progesterone inhibits LH secretion in males (47), it remains unclear whether this occurs through direct inhibition of pulse generator activity, as observed in females.

Furthermore, the sexually dimorphic aromatase neuronal network, which converts testosterone to estrogen, plays a role in regulating ARN^KISS^ neuronal activity in males (48). It is also unknown whether an identical hormonal environment would lead to sex-specific differences in negative feedback through ARN^KISS^ downstream signaling pathways.

The greater suppressive effect of gonadal steroids in male pulse generator activity may reflect a more straightforward on–off regulatory mechanism that supports continuous sperm production, whereas females require more complex and nuanced hormonal regulation to accommodate the complexities of the reproductive cycle.

### Gonadal Steroid-independent Differences in GnRH Pulse Generator

Although the main profiles of synchronization and activity patterns in males and females were mostly similar after gonadectomy, subtle sex differences were observed in the SI interval distribution. This suggests the involvement of steroid-independent factors in regulating pulse frequency. These differences may arise from intrinsic electrophysiological properties, as evidenced by sex-specific firing patterns in brain slices (49). Additionally, afferent connections to ARN^KISS^ neurons exhibit sexual differences, potentially leading to distinct modulation of kisspeptin neuron activity (50). Females have also been shown to display greater responsiveness to neuropeptides such as vasoactive intestinal peptide (51).

Recent GCaMP6 studies in vitro suggest sex differences in the role of glutamate and neurokinin B, along with a 58% higher miniature SE frequency in females compared to males (12, 22). This finding points to an intrinsically higher excitability in females, further supported by recordings of spontaneous firing in brain slices (49). Moreover, females exhibit lower capacitance (22, 52) and a higher resting membrane potential (22), both of which contribute to increased neuronal excitability. It is also important to consider the potential influence of differential gonadal steroid programming during development, as documented in the anteroventral periventricular nucleus kisspeptin neurons (53, 54). This may also apply to the ARN kisspeptin neurons, where a difference in their synchronization frequency persists independently of gonadal steroids in adulthood.

In conclusion, this study demonstrates that sex differences in the operation of the ARN^KISS^ neuron pulse generator are largely determined by circulating levels of gonadal steroids. Future experiments directly examining the changes in the recruitment mechanism of individual ARN^KISS^ neurons during synchronization in intact and GDX states of both sexes will provide further insight into the sex- and gonadal state-dependent mechanisms regulating the GnRH pulse generator.

## Data Availability

Data supporting the findings of this study are available from the corresponding author on reasonable request.
